# Sex Hormones and Satellite Cell Regulation in Women

**DOI:** 10.1155/2022/9065923

**Published:** 2022-04-14

**Authors:** Mikkel Oxfeldt, Line Barner Dalgaard, Jean Farup, Mette Hansen

**Affiliations:** ^1^Department of Public Health, Aarhus University, Aarhus, Denmark; ^2^Department of Biomedicine, Aarhus University, Aarhus, Denmark; ^3^Steno Diabetes Center Aarhus, Aarhus University Hospital, Aarhus, Denmark

## Abstract

Recent years have seen growing scholarly interest in female physiology in general. Moreover, particular attention has been devoted to how concentrations of female sex hormones vary during the menstrual cycle and menopausal transition and how hormonal contraception and hormonal therapy influence skeletal muscle tissue. While much effort has been paid to macro outcomes, such as muscle function or mass, rather less attention has been paid to mechanistic work that may help explain the underlying mechanism through which sex hormones regulate skeletal muscle tissue. Evidence from animal studies shows a strong relationship between the female sex hormone estrogen and satellite cells (SCs), a population of muscle stem cells involved in skeletal muscle regulation. A few human studies investigating this relationship have been published only recently. Thus, the purpose of this study was to bring an updated review on female sex hormones and their role in SC regulation. First, we describe how SCs regulate skeletal muscle maintenance and repair and introduce sex hormone signaling within the muscle. Second, we present evidence from animal studies elucidating how estrogen deficiency and supplementation influence SCs. Third, we present results from investigations from human trials including women whose concentrations of female hormones differ due to menopause, hormone therapy, hormonal contraceptives, and the menstrual cycle. Finally, we discuss research and methodological recommendations for future studies aiming at elucidating the link between female sex hormones and SCs with respect to aging and training.

## 1. Introduction

In the past decade, a growing body of evidence has confirmed that satellite cells (SCs) are important for regulation and maintenance of skeletal muscle mass. Moreover, they are essential for muscle regeneration [1–3] and may support skeletal muscle hypertrophy when performing resistance training. SCs are controlled by complex cellular and molecular interactions that remain incompletely understood [3]. Located in close proximity to the muscle fiber between the sarcolemma and the basement membrane, most SCs are only a few micrometers away from a blood vessel [4]. This unique location makes SCs able to respond promptly to signals both from the muscle fiber itself, such as mechanical tension, and from changes in the local and systemic environment, such as hormonal fluctuations [5, 6].

In women, the majority of sex steroid hormones are produced in the adrenals and ovaries and then delivered to peripheral tissues by circulation to promote endocrine effects. Yet, several tissues express enzymes capable of local tissue synthesis of steroid hormones, including skeletal muscle [7, 8], which has been demonstrated both *in vitro* and *in vivo* [8, 9]. The latter nongenital synthesis of sex hormones becomes relatively more important during aging and after menopause, when the secretion of sex hormones from the genitals decreases markedly. In men, exercise is shown to be one mode of action to activate local steroidogenesis after both short- and long-term training programs [10, 11]. In line with this observation, resistance training stimulates SC activity [12–14], and fluctuation in systemic estrogen seems to directly influence SC abundance [15].

Estrogen is the predominant female sex hormone together with progesterone, luteinizing hormone (LH), and follicle-stimulating hormone (FSH). Before puberty, sex hormone levels are generally low. The reproductive stage of life is characterized by cyclic rises and falls in endogenous sex hormone levels until the transition into menopause, which is marked by decreases in estrogen and progesterone levels and increases in FSH and LH. The changes in the sex hormones throughout the female life cycle constitute one of the major differences between female and male physiology. This has raised speculations about the impact of the hormones and hormonal changes on various physiological parameters related to regulation of skeletal muscle mass and physical performance. For instance, increasing age negatively influences skeletal muscle. However, women also seem to experience an accelerated loss of muscle strength and mass during the transition into menopause, which is paralleled by a marked decline in circulating estrogen [16–20]. This suggests that estrogen plays a role in maintaining muscle mass and function.

A number of excellent review papers have previously discussed the role of sex hormones on muscle regeneration and SCs, drawing primarily on data from *in vitro* and animal studies [15, 21]. However, recent years have seen a number of human trials, warranting the need for an updated review. The majority of studies have focused on the role of estrogen, while the impact of progesterone remains mostly unknown. The aim of this study was to review the existing literature investigating the relation between SCs and estrogen and other sex hormones in animal and human trials.

## 2. Background

### 2.1. SC Activation and Their Importance for Skeletal Muscle Regeneration and Hypertrophy

SCs are a population of stem cells that under resting conditions in adults are quiescent. In response to stimuli such as skeletal muscle injury or muscle growth signals, SCs are activated, enter the cell cycle, and begin to proliferate. SCs are essential for muscle regeneration [1, 2] and undergo asymmetric division to produce daughter cells that either return to quiescence to repopulate and preserve the SC pool [22, 23] or enter the myogenic program to differentiate and fuse to form new myofibers during myofiber regeneration. Alternatively, SCs can fuse with existing myofibers during muscle hypertrophy following muscle overload models or resistance exercise [24–26]. Committed to the myogenic program, SCs migrate to the site of injury, where a complex orchestrated cellular response ensures proliferation, differentiation, and fusion with new and existing myofibers (for a detailed review, see Dumont et al. 2015 [3]).

**Figure 1:**
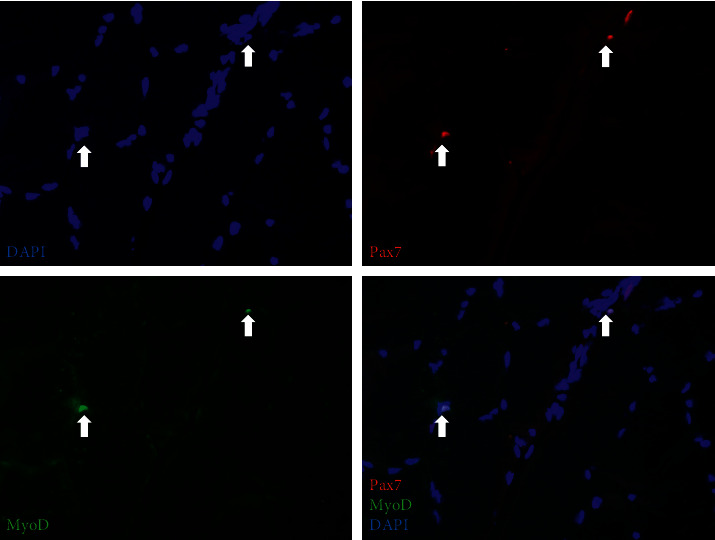
Representative images of immunohistochemical staining of SCs in muscle from a healthy young male subject: nuclei (blue/DAPI), pax7 (red), and MyoD (green). The merged image shows co-localization of Pax7 and MyoD with white arrows indicating DAPI+/Pax7+/MyoD+SCs.

Today, SCs are often identified with immunohistochemical staining, by which the unique cellular location of SCs and detection of cell-specific proteins can be combined (Figure 1). Both quiescent and activated SCs uniquely express the transcription factor-paired box protein 7 (PAX7), making PAX7 an excellent marker to describe and quantify SCs in skeletal muscle. Co-identification of PAX7 together with Ki-67 or proliferating cell nuclear antigen (PCNA) can identify proliferating SCs. At later stages of regeneration, myogenic commitment and differentiation can be recognized by the expression of myogenic transcription factors, such as MyoD, and later myogenin and MRF4.

It is well documented that SCs are vital for postnatal muscle development [27–29] and adult muscle regeneration [1, 2]. Animal models with conditional depletion of adult skeletal muscle SCs demonstrate that elimination of SCs impairs the muscles' ability to regenerate from injury [30]. Remarkably, as few as seven SCs associated with one transplanted myofiber can generate over 100 new myofibers containing thousands of myonuclei [31, 32]. Despite this, the role of SCs in skeletal muscle growth is still debated [33]. Early studies, using irradiation to impair SC function, suggested a need for SCs in muscle hypertrophy [34], which was confirmed by later studies suggesting that SC fusion with existing myofibers precedes muscle hypertrophy [24]. The early data indicating a need for SC-derived myonuclei for muscle hypertrophy provided the foundation for the myonuclear domain theory [35]. This theory suggested that a myonuclei can only provide transcriptional support for a finite myofiber volume and that hypertrophy above a certain threshold would require new myonuclei to increase the transcriptional capacity of the myofiber [36]. In contrast to this hypothesis, recent studies using more specific genetic strategies for SC ablation have shown that SC depletion does not impair short-term hypertrophic capacity [26]. However, follow-up studies have later confirmed that SCs seem to play an important role in muscle hypertrophy over a prolonged training period [25, 37] as well as in other measures of muscle function [38–40]. These data are supported by longitudinal training studies in humans, in which an increase in SC content is tightly associated with muscle hypertrophy [41–43]. Furthermore, high responders to resistance training are characterized by a high basal SC content compared to low responders and experience greater increases in SC and myonuclei number in response to resistance training [44]. Hence, human data seem to support a role for SCs in skeletal muscle hypertrophy. It should be noted that non-hypertrophic exercise can also increase the SC content [45], indicating that expansion of the SC pool may rely on other mechanisms than those related to myofiber size and myonuclear domain.

### 2.2. Female Sex Hormones and Receptors in the Skeletal Muscle

The female sex hormones, estrogen and progesterone, are steroid hormones that execute their actions through binding to receptors present on the extracellular side of the basement membrane or bypass the phospholipid-rich sarcolemma to interact with receptors or molecules intracellularly within the cytosol or the nuclei [46]. The identification of new hormone receptors is an ongoing process, which continually adds new candidates to the group of known steroid hormone receptors as research develops.

The primary binding sites for estrogen are the estrogen receptor (ER)-alpha and -beta (ER-*α*, ER-*β*), localized primarily in reproductive tissues, and to a lesser extent in other tissues such as the cardiovascular system, adipose tissue, brain, bone [47], and within skeletal muscle tissue [48]. Estrogen is primarily considered to function by binding to cytosolic ERs, resulting in the translocation of the ligand/receptor complex to the nucleus and binding to a 13-bp palindrome sequence, known as the estrogen response element (ERE) [49]. Following binding, the newly formed ER-ERE complex is able to activate the transcription of estrogen responsive genes, also known as genomic actions [50]. However, ERs are also located on the extracellular membrane, e.g., the G-protein-coupled ER (GPER), where estrogen-ER binding may directly activate rapid (nongenomic) intracellular signaling cascades and influence signal transduction proteins [49, 50]. Finally, estrogen may also be able to act independently of estrogen receptors to induce cellular signal transduction [15].

17-*β*-estradiol is the most potent and abundant form of endogenously produced estrogen. Therefore, 17-*β*-estradiol is the most widely investigated isoform of estrogen. However, today millions of women worldwide consume exogenous sex hormones through hormone replacement therapy (HRT) or as oral contraceptives. While many HRT products deliver a synthetic produced 17*β*-estradiol, oral contraceptives contain estrogen in the form of 17*α*-ethinyl estradiol. The addition of the ethinyl group produces an estrogen variant with a longer half-life and a higher binding affinity to the ERs [51, 52]. The pharmacokinetic profile of these different types of estradiol varies, and their presence is also influenced by other external and internal factors, such as smoking and genetic heritage [53, 54]. However, very little is known in terms of how these synthetic estrogen variants influence skeletal muscle homeostasis and cellular signaling.

The progesterone receptor (PR) exists in two isoforms, PRA and PRB that form both homo- and heterodimers [55, 56]. Like the ERs, PR is primarily present in reproductive tissues but is also detected in other tissues such as skeletal muscle [57]. PRB is a positive promotor of progesterone effects, while PRA functions to antagonize the PRB effects as well as ER effects [58]. Several studies indicate that activation of the PR gene depends on estrogen binding the ER [56, 59], but also non-estrogen-dependent actions, e.g., cyclin D1 binding to the ER [60]. Therefore, physiological responses to progesterone may be regulated by the combined effects of both progesterone and estrogen acting via their respective receptors.

**Figure 2:**
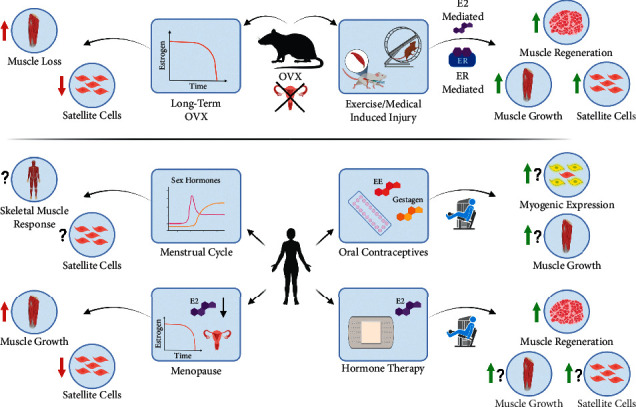
Visual summary of the current body of evidence elucidating the influence of female sex hormones on SC regulation. Red arrows indicate a negative effect. Green arrows indicate a positive effect. Question marks indicate that the evidence is sparse and possibly inconclusive. OVX = ovariectomized; ER = estrogen receptor; *E*2 = 17*β*-estradiol; EE = ethinyl estradiol. Created with Biorender.com.

Whether SCs express steroid hormone receptors themselves and/or SCs are regulated by estrogen via genomic or rapid nongenomic receptor binding in muscle fibers or other types of cells within the skeletal muscle tissue is still not fully elucidated. In the next section, we will discuss the influence of or lack of estrogen and the ERs on SCs in animals at rest and in response to exercise in more detail. We attempt to present the findings chronologically as new studies are developed and built upon previous observations. A visual summary of the body of evidence elucidating the influence of female sex hormones on SC regulation in both animals and humans is presented in Figure 2.

## 3. Animal Studies

### 3.1. Influence of Estrogen and ERs on SCs

In 2005, Tiidus et al. demonstrated for the first time that estrogen supplementation to male rats significantly increased SC numbers in both type I and type II skeletal muscle fibers following three days of downhill running [61]. These findings were reported following a number of studies observing lower myofibrillar damage, reduced release of markers for muscle damage (creatine kinase), and reduced inflammatory response after eccentric exercise in female rats compared to male rats and estrogen-deficient ovariectomized (OVX) rats [62, 63]. Since then, a number of papers have been published elucidating the mechanisms by which estrogen influences SC regulation. Enns et al. investigated which stages of the SC cycle they were influenced by estrogen supplementation in control or OVX rats [64]. Here, they quantified total SC content (PAX7-positive) as well as proliferating MyoD-positive and 5-bromo-2′-deoxyuridine (BrdU)-positive SCs 72 hours after downhill running. Postexercise, estrogen-treated OVX female rats had greater SC content as well as increased SC proliferation compared to OVX rats. This suggests that several stages of the SC cycle may be augmented by estrogen, possibly via event upstream of SC activation [64]. Data by Larson et al. further support estrogen's influence on SCs. They investigated how repeated injuries to OVX and 17*β*-estradiol-supplemented mice impacted recovery and SC number [65]. SCs in TA muscles were quantified by the use of flow cytometry (CD45−/CD31−/VCAM1+/*α*7 integrin + cells); the results showed that repeated BaCl_2_-induced injury reduced the number of SCs, and this reduction was not significantly altered by 17*β*-estradiol supplementation. However, when SCs were quantified three times from weeks 3 to 12 after ovariectomy, the SC number was ∼42% lower in 17*β*-estradiol-deficient mice than in 17*β*-estradiol-supplemented mice. In addition, 17*β*-estradiol supplementation resulted in superior gains in muscle mass and strength over the 12-week experimental period compared to OVX mice [65]. This demonstrates that estrogen deficiency blunts regenerative processes in skeletal muscle and that this is likely due to a reduction in the number of SCs.

An estrogen's positive effect on SC regulation is possibly mediated through the ERs. Accordingly, ER-*α* and ER-*β* are expressed in mouse SCs [66], and in 2008, Enns et al. demonstrated that blocking both the ER-*α* and ER-*β* receptors completely abolished exercise and 17*β*-estradiol-mediated increases in SC markers (PAX7, MyoD, BrdU) determined by immunohistochemistry [67]. This suggests an important role of the ERs in SC regulation. However, a notable observation by the authors was that 17*β*-estradiol-induced attenuation of muscle damage and leucocyte infiltration was unaffected by the ER antagonist. The latter may relate to estrogen having a stabilizing effect on the muscle membrane [63] and thereby reduces the damage and subsequent need for leucocyte infiltration independently of the ERs. The role of the ERs in SC regulation was further examined in a follow-up study by Thomas et al., specifically investigating the role of ER-*α* in SC activation. Here, they used the ER-*α* agonist; propyl pyrazole triol alone and in combination with 17*β*-estradiol supplementation to OVX rats. Interestingly, they observed that specific stimulation of ER-*α* increased the SC content and activation to the same extent as 17*β*-estradiol alone or combined 17*β*-estradiol with propyl pyrazole triol [68]. Based on these results, the authors concluded that estrogen acts through an ER-mediated mechanism to stimulate SC proliferation following exercise, with ER-*α* playing a primary role. In support of this proposed mechanism, Collins et al. recently demonstrated through RNA sequencing and qRT-PCR that isolated SCs express significantly more ER-*α* than ER-*β*, Gper1, and PR [69]. They also observed comparable reductions in the SC pool in ER-*α* knockout mice and OVX mice compared to wild type [69]. Combined, these data indicate that ER-*α* plays an important role in the estrogen-mediated regulation of SCs.

ER-*β*s involvement in the estrogen-SC interaction was first studied in animals by Velders et al. [70]. In their comprehensive study, animals were injured by notexin and the influence of ER-*α* and ER-*β*-selective ligands in OVX rats and also ER-*α* and ER-*β* knockout mice was investigated. Interestingly, knockout of ER-*β* was associated with a reduced ability to induce PAX7 mRNA expression and greater elevation in creatine kinase after notexin-induced muscle injury. Furthermore, MyoD mRNA expression was significantly decreased in the injured skeletal muscle in ER-*β* knockout mice compared to ER-*α* knockout mice and wild-type mice, suggesting that loss of ER-*β* negatively affects the skeletal muscle regenerative processes. Finally, cell cycle activity determined by PCNA mRNA and myogenic determination by MyoD mRNA were significantly increased by 17*β*-estradiol and the ER-*β* selective ligand, but not the ER-*α* ligand, in OVX rats compared to OVX rats receiving placebo. Notably, neither PCNA nor MyoD are SC specific [70]. Nevertheless, the authors proposed that ER-*β* signaling is involved in the regulation of skeletal muscle growth and regeneration by stimulating anabolic pathways and activating SCs. In support of ER-*β* playing an important role during muscle regeneration, Seko et al. reported hampered muscle regeneration in injured ER-*β* knockout mice compared to control mice, and this was coupled to a lower SC number and a greater muscle loss [71]. *In vitro* experiments further demonstrated that ER-*β* deletion resulted in decreased expression of Ccna2, a cell-cycle regulator, and concomitant impairment of SC proliferation. Notably, in contrast to the injured muscle, there was no effect of ER-*β* knockout on the maintenance of muscle mass or cross-sectional area under homeostatic conditions.

To sum up, the abovementioned studies provide strong evidence for estrogen playing an important role in skeletal muscle regeneration through SC regulation following exercise or chemically induced muscle injury (Figure 2). Current evidence suggests that several stages of SC regulation are influenced by estrogen and the regulation is likely ER-mediated. Animal models indicate that stimulation of both ER-*α* and ER-*β* play important roles in mediating the positive estrogenic stimulating effect on SCs, with ER-*α* likely being the ER important for maintaining the SC pool [69], and ER-*β* possibly playing a role for muscle regeneration in response to injury [70, 71]. Given the loss of SCs following ablation of ER-*α*, future studies should aim to investigate the SC cell fate when estrogen is lacking.

### 3.2. Consequences of Estrogen Deficiency on SCs in Animals

To understand how estrogen deficiency influences SCs, Kitajima et al. examined how OVX of mice affected SCs compared to sham-operated mice. Interestingly, OVX did not result in an altered content of SCs compared to control animals when measured 8 weeks after ovariectomy [72]. Moreover, a long-term follow-up study by the same research group reported no difference in SC content in isolated single myofibers in female mice 24 weeks after OVX [73]. However, following three days of single fiber culture, they observed reduced SC differentiation (PAX7−/MyoD+) and indices of impaired self-renewal (PAX7+/MyoD−) in SCs from the OVX mice. Collectively, these findings indicate that OVX, and thereby a reduced estrogen level, impairs SC function during activation of the SC pool.

In contrast to the findings of Kitajima and Ono [73] showing no change in SC content after ovariectomy under resting conditions, other studies have found the SC pool to decrease after ovariectomy [69, 74]. For instance, using flow cytometry, Frechette et al. demonstrated that six weeks after ovariectomy, OVX mice experienced a significant decrease in the content of SCs (percentage CD45−/CD32−/SCA1−/integrin-a7+/CD34+) [74]. In support, Collins et al. recently reported a 30%–60% decline in SC numbers in the tibialis anterior muscles of OVX mice at rest [69]. The decline in SC number was associated with the time since OVX and thereby time with sex hormone deficiency (2, 4, and 7 months) [69]. Similar results were reported for extensor digitorum longus, gastrocnemius, and diaphragm at rest, but not the slower and more fatigue resistant soleus muscle [69]. Furthermore, histological analysis of PAX7+ cells revealed 50% fewer SCs in the tibialis anterior muscle from the OVX mice compared to controls, whereas treatment with 17*β*-estradiol rescued SC numbers in OVX mice and thereby prevented depletion of the SC pool [69].

In summary, animal findings suggest that prolonged estrogen deficiency reduces SC number and SC quality by means of expansion, differentiation, and maintenance (Figure 2). It remains unclear what the underlying mechanism by which deficiency of estrogen (or related hormones) affects SCs. In particular, the loss of SCs under resting conditions (i.e., without prior muscle injury) is likely to be relevant in humans, particularly the aging population. Thus, understanding the mechanisms by which estrogen governs SC turnover and maintenance will be important to potentially prevent this unfortunate decline in SCs and the related consequences that come with it.

The following sections will focus on the evidence that is available from human studies regarding the influence of estrogen and related sex hormones on human SCs (Figure 2). Although the evidence from human studies is sparse, this is an area of increasing interest that deserves more attention.

## 4. Human Studies

### 4.1. Consequences of Aging and Estrogen Deficiency on SCs in Women

A decline in muscle strength and mass occurs with age in both men and women [75]. The decline in strength is partly caused by age-induced muscle atrophy, but the Baltimore Longitudinal Study on Ageing documented that muscle quality (specific strength normalized to muscle size) also declines with age [76]. Age-dependent muscle atrophy is likely a multifactorial process dependent on physical activity level, low grade inflammation status, and a range of metabolic processes, but it is likely also influenced by sex hormonal changes during aging [77].

The transition into menopause in women represents a unique physiological model; in a relatively short time span, women shift from cyclic fluctuations in estrogen and progesterone every month to an essentially sex hormone-depleted state [78]. As such, the menopausal transition has been used to study estrogen deficiency in women, although it can be difficult to separate the effects of estrogen deficiency from the effects of aging.

Studies have indicated an accelerated loss of muscle strength and mass around the age of menopause [16, 17, 20]. The SC number has been shown to be reduced in old men compared to young men [79–81], but how the SC pool is influenced by age and the transition into menopause in women is less studied. A small but well-conducted study by Collins and colleagues recently investigated the changes in SC number during the menopausal transition in muscle tissue collected during perimenopause and early postmenopause from five women [69]. They showed a borderline significant decline (15%, 4/5 women) in SC number within one year, and the percentage of PAX7+ cells correlated positively with individual serum 17*β*-estradiol levels. Furthermore, this decline was not affected by changes in physical activity or myofiber size, as these parameters did not change significantly within the one-year time span. While these findings are based on a limited number of participants (*n* = 5) and need to be replicated in larger scales, they mimic findings from animal studies providing a negative relationship between estrogen deficiency and SCs.

### 4.2. The Effect of Sex Hormone Therapy on Skeletal Muscle and SCs in Aging Women

A promising but still controversial strategy to fight the loss of muscle mass and strength in postmenopausal women is to use hormone replacement therapy (HRT) containing a combination of estrogen and progesterone. The most convincing evidence today of a positive effect of HRT was provided by the Finnish Twin Cohort [82] investigating fifteen 54- to 62-yr-old monozygotic female twin pairs, one sister being HRT user and the other being nonuser. In this study, HRT was associated with significantly higher maximal 10-m walking speed and muscle power in the lower body. Interestingly, it was also reported that the HRT users had greater relative muscle area and less relative fat area of the thigh than twin sisters not using HRT. To the best of our knowledge, no human data have been published examining the influence of estrogen deficiency vs. estrogen treatment (i.e., HRT-users vs. nonusers) on SCs. Nevertheless, Dieli-Conwright and colleagues demonstrated that in response to a bout of high intensity resistance exercise, HRT users experienced less muscle damage, but greater increases in myogenic regulatory factors compared to nonusers [83, 84]. These findings indicate that postmenopausal women using HRT may have a greater myogenic potential to facilitate a greater myogenic response to exercise. In line with these findings, we have observed a higher myofibrillar protein fractional synthesis rate 24 hours after resistance exercise in 10 women who had undergone hysterectomy (and 9/10 oophorectomy) and were on estrogen therapy, whereas in 10 age-matched postmenopausal controls, the myofibrillar protein fractional synthesis rate did not differ between the exercise leg and the leg, which had rested [85]. Furthermore, in a double-blind controlled intervention study, we recently observed that early postmenopausal women who randomized to wear transdermal estrogen patches during 12 weeks of resistance training showed increased muscle cross-sectional area and whole-body fat-free mass to a greater extent than women-wearing placebo patches during the training period [86]. Interestingly, a significant time × treatment interaction was observed in the number of PAX7-positive SCs detected by immunohistochemistry in type I fibers with a numerical increase and decrease in the estrogen and placebo groups, respectively (*data submitted*).

Importantly, the timing of HRT treatment relative to menopause may affect the potential benefits. Some evidence exists demonstrating that skeletal muscle ER protein content is lower in late postmenopausal women compared to that in early postmenopausal women [87]. Furthermore, short-term supplementation with 17-*β*-estradiol reduces skeletal muscle protein breakdown markers in early but not late postmenopausal women [88], suggesting that effects of 17-*β*-estradiol supplementation on skeletal muscle depend on time since menopause. This finding was verified by an animal study by Mangan et al. [89] elucidating whether an 11-week delay in 17-*β*-estradiol replacement after ovariectomy would affect the ability of estrogen to augment postexercise muscle SC proliferation in rats. Interestingly, they observed that if estrogen replacement was delayed after postovariectomy, the stimulating effect of estrogen on SC proliferation in response to exercise was lost. In perspective, these latter results are important when listing up the pros and cons regarding initiating the use of HRT after menopause.

### 4.3. The Skeletal Muscle and SC Response to Oral Contraceptives in Premenopausal Women

All over the world, many women use hormonal contraceptives, with oral contraceptives (OCs) being the preferred form of contraception in young women [90]. In Denmark, 57% of elite female athletes use hormonal contraceptives, with 73% using OCs [91]. OCs contain synthetic estradiol (ethinyl estradiol, EE) and one type of progestin (a synthetic form of the body's naturally occurring hormone progesterone). The exogenous administration of sex hormones suppresses the endogenous secretion of sex hormones and thereby change the sex hormonal profile. How these synthetic hormones influence skeletal muscle has only been studied to a limited extent. Nevertheless, a number of studies have reported differences in muscle recovery and adaptation to training between users of OCs and eumenorrheic women [92–94]. However, based on the available literature, it is difficult to draw a conclusion on how OCs influence skeletal muscle, since the results are conflicting which may relate methodological limitations, with small sample sizes and lack of differentiation between types and doses of OCs being the main concerns.

To better understand how different OCs influence adaptations to training in skeletal muscle, our research group conducted two longitudinal training studies. Here, we examined the response to resistance training in 3^rd^ generation OC users [93], the most commonly used OC in Australia [95], and in 2^nd^ generation OC-users [94], the most commonly used OC in Nordic countries [90]. Interestingly, we observed that 3^rd^ generation OC-users experienced a tendency (*p*=0.06) toward greater improvement in muscle cross-sectional area and a significantly greater improvement in type I muscle fiber CSA compared with nonusers of OCs following 10 weeks of resistance training [93]. In the 2^nd^ generation OC-users, we found comparable gains in muscle cross-sectional area between groups, but a tendency (*p*=0.08) toward a greater increase in whole-body fat-free mass in the OC-users compared to controls [94]. Furthermore, when we analyzed muscle samples collected from the latter study for molecular markers of skeletal muscle hypertrophy, we observed a significant increase in SC number per total fiber and the SC number per type II fiber in the OC-users only [96]. In addition, analyses of resting mRNA levels revealed that the expression of MRF4 mRNA was significantly increased in OC-users compared to controls after the training intervention. Collectively, these studies may suggest that OC-users experience a greater anabolic response to resistance training due to OC use, triggering a greater myogenic potential [96]. We have been unable to identify other published human studies investigating SC abundance or myogenic expression in OC-users. Nevertheless, a recently published in vitro study using skeletal muscle myoblast cultures, found that exposure to physiological levels of circulating hormone concentrations of ethinyl estradiol and the progestin Dienogest significantly increased myogenin, PCNA, and follistatin protein expression and decreased myostatin protein expression [97]. These data support a promyogenic effect of the synthetic hormones administered through OCs. However, future in vivo studies are needed to confirm if OC use amplifies muscle growth during training and clarify how synthetic sex hormones promote myogenic proliferative proteins in comparison with endogenous estrogen and progesterone.

### 4.4. Influence of the Menstrual Cycle on the Skeletal Muscle Response to Exercise

As we have previously addressed in this review, animal studies have found that ERs are important for estrogen's positive effect on SC regulation. Hence, it could be hypothesized that the myogenic potential during the menstrual cycle is ensured in young women by regulation of the expression of ERs when the level of estrogen and progesterone fluctuates. While previous research primarily has focused on fluctuations in serum hormone levels during the menstrual cycle, a recent study by Ekenros et al. [57] elucidated the mRNA and protein levels of sex steroid receptors in skeletal muscle tissue in 15 eumenorrheic women at three time points during the menstrual cycle; in the early follicular phase, the late follicular phase, and the mid luteal phase. A significant variation in mRNA and protein levels of ER-*α* and PR was found across the menstrual cycle. ER-*α* mRNA and protein were highest in the early follicular phase when estradiol was low, whereas PR protein expression was highest in the luteal phase where both the levels of estradiol and progesterone were relative high. In another study by Haines et al., 22 eumenorrheic women completed two eccentric exercise protocols during each phase of the menstrual cycle (day 6 for mid-follicular and day 21 for mid luteal) [98]. Here, ER-*α* mRNA and protein expression were also observed to be greater in the mid-follicular phase, while ER-DNA binding and MyoD mRNA expression increased in response to eccentric exercise independently of the menstrual cycle phase. These data are interesting, since an increase in ER-DNA binding indicates increased estradiol-ER binding and subsequent ERE binding (ER-ERE complex), and this was observed concomitantly with enhanced MyoD mRNA expression and may indicate an ER-dependent activation of MyoD [98]. In the same study, cyclin D1, a multifunctional G_1_-phase cyclin able to activate ER-mediated transcription of estradiol-responsive genes, increased in response to exercise, but to a significantly greater extend in the mid-follicular phase compared to the mid luteal phase [98]. The authors proposed that low levels of estradiol during the mid-follicular phase may preferentially increase the expression of cyclin D1 to counteract the lack of estradiol and thereby ensure responsiveness in MyoD expression to exercise both when estradiol is high and low.

Much work is still needed in this area of research. The limited evidence that exists suggests that muscle regeneration, sex steroid receptors, and estrogen signaling fluctuates just like sex hormones over the menstrual phases (although in an opposite direction of serum estrogen levels), why SC regulation is most likely influenced as well. However, to understand how these physiological processes intercept, more research should investigate the molecular and metabolic responses to the menstrual cycle.

## 5. Recommendations for Future SC Research

### 5.1. Research Directions for Human Studies

Today, animal studies clearly demonstrate that prolonged estrogen deficiency reduces SC number and SC quality and that estrogen supplementation can augment SC number and SC responsiveness and thereby promote skeletal muscle regeneration in response to injury (Figure 2).

As we have covered in this review, the number of human studies elucidating the regulatory role of sex hormones on SCs is still limited (Figure 2). Nevertheless, most convincingly, studies comparing HRT-users to nonusers have shown lower muscle damage and greater increases in myogenic regulatory factors in response to an acute resistance exercise bout, and 12 weeks of resistance training resulted in greater gains in muscle mass. However, still we need to gain more knowledge within this field regarding (1) the mechanistic processes (including the role of SCs) causing a deterioration of skeletal muscle mass and function during the transition into menopause. Future studies should collect muscle samples from perimenopausal vs. postmenopausal women or ideally monitor the same women through the menopausal transition and analyze the samples for changes in SCs and regulatory muscle protein markers in perspective to changes in muscle mass. (2) How does hormone supplementation effect SCs in postmenopausal women? We encourage new randomized controlled double-blind studies to use postmenopausal women as a good and feasible model to examine the impact of estrogen/hormone supplementation on SC content and related skeletal muscle mass and function in response to anabolic stimuli such as training. (3) How does the administration of different types of hormonal contraceptives to premenopausal women influence skeletal muscle mass and function in general and in response to resistance exercise training, and which role do SCs play in this context? Randomized controlled double-blind studies are needed to elucidate the causality between the use of hormonal contraceptives and changes in skeletal muscle mass in general and in response to supervised training interventions. (4) How does fluctuations in sex hormones during the menstrual cycle impact the SCs and other relevant parameters related to sex hormone signaling such as the hormone receptors? We encourage future studies to elucidate the regulatory role of SCs in skeletal muscle by comparing different phases using high quality methodology to define the menstrual phases [99].

### 5.2. Methodical Considerations for SCs in Human Studies

Studies on SCs in human skeletal muscle have traditionally focused on SC quantification using immunohistochemistry, potentially combined with a cell activity marker such as KI67, PCNA, or MyoD to confirm SC activation [100]. Using this methodology, researchers can explore the SC response to endocrine and external (e.g., exercise) stimuli. SC content can also be explored using whole muscle gene or protein expression of SC specific proteins such as PAX7. Such data should, however, be interpreted with caution since subsets of SC/progenitor cells may have different expression of, e.g., PAX7 [101]; hence, changes in PAX7 expression are not necessarily related to an increased quantity of SCs per se. Examining SC activity using whole muscle gene or protein expression is also challenged by the lack of SC specificity of many of these targets (e.g., KI67 and PCNA). Given the large quantity of other non-SCs in human muscle [102], the increased whole muscle expression of such proliferation markers could originate from many other cell types. Therefore, when performing studies on women, we recommend that researchers, at minimum, consider collecting skeletal muscle biopsies for immunohistochemistry and the possibility for quantification of SCs. It is, however, worth considering that the *in vivo* SC cell cycle activity, and thus the expression of proliferation markers, is generally very low under homeostatic conditions [45, 103]. Recent studies in ageing mice have revealed that the speed at which SCs enter the cell cycle (time-to-first division) upon activation (e.g., following injury) is critical for proper regeneration [104, 105]. Moreover, this time-to-first division is prolonged in aged SCs, resulting in delayed regeneration [105]. With the recent developments in direct SC isolation from human skeletal muscle, this opens the possibility to assess time-to-first division in human SCs [102, 106]. Future studies should therefore not only investigate changes in SC content but also the potential functional impairment of SCs (e.g., time-to-first division) as a consequence of the loss of estrogen, as suggested by animal data [69]. This could also reveal potential defects in the regulation of stem cell quiescence as a consequence of the hormonal changes at menopause.

In addition to the more advanced understanding of SC function, the recent development of single cell, single nuclei, and spatial transcriptomics has paved the way for a better insight into SC dynamics, also in human skeletal muscle [102, 107, 108]. Utilizing such methodologies will provide the framework for better insight into the mechanisms underlying SC dysfunction as a consequence of loss of estrogen.

## 6. Conclusion

Animal studies provide strong evidence for an estrogen's role in promoting skeletal muscle regeneration through SC regulation in response to injury. In line with this, prolonged estrogen deficiency reduces both SC number and quality by means of expansion, differentiation, and maintenance of the SC pool. Overall, there is evidence to suggest that both the former and the latter are mediated through the ERs. Studies in humans point in the same direction, although mainly documented in postmenopausal women with or without hormone supplementation, which mimics the OVX animal model (i.e., a scenario of hormonal polarity). Moreover, evidence suggests that the transition into menopause may cause a rapid drop in the SC pool, while hormone therapy promotes skeletal muscle regeneration and possibly adaptations to resistance training. In premenopausal women characterized by hormonal fluctuations during the menstrual cycle or a changed hormonal milieu due to the use of hormonal contraceptives, the evidence is still limited and results inconclusive in regard to the role of SCs. Despite that a limited number of human trials are available, the data are so far compelling. Therefore, we encourage future human trials to further investigate the interaction between female sex hormones and SCs.

## Data Availability

No data were used to support this study.
